# Associations of genetically predicted IL-6 signaling with cardiovascular disease risk across population subgroups

**DOI:** 10.1186/s12916-022-02446-6

**Published:** 2022-08-11

**Authors:** Marios K. Georgakis, Rainer Malik, Tom G. Richardson, Joanna M. M. Howson, Christopher D. Anderson, Stephen Burgess, G. Kees Hovingh, Martin Dichgans, Dipender Gill

**Affiliations:** 1 Center for Genomic Medicine, Massachusetts General Hospital, Richard B. Simches Research Center, 185 Cambridge Street, CPZN 6818, Boston, MA 02114 USA; 2 Program in Medical and Population Genetics, Broad Institute of Harvard and the Massachusetts Institute of Technology, Boston, MA USA; 3 Institute for Stroke and Dementia Research (ISD), University Hospital, Ludwig-Maximilians-University (LMU) Munich, Munich, Germany; 4 Genetics Department, Novo Nordisk Research Centre, Oxford, UK; 5 Department of Neurology, Brigham and Women’s Hospital, Boston, MA USA; 6 Medical Research Council Biostatistics Unit, University of Cambridge, Cambridge, UK; 7 Cardiovascular Epidemiology Unit, Department of Public Health and Primary Care, University of Cambridge, Cambridge, UK; 8 Department of Vascular Medicine, Academic Medical Center, Amsterdam University Medical Centers, University of Amsterdam, Amsterdam, The Netherlands; 9 Global Chief Medical Office, Novo Nordisk, Copenhagen, Denmark; 10 Munich Cluster for Systems Neurology (SyNergy), Munich, Germany; 11 German Centre for Neurodegenerative Diseases (DZNE), Munich, Germany; 12 Department of Epidemiology and Biostatistics, School of Public Health, Medical School Building, St Mary’s Hospital, Imperial College London, London, W2 1PG UK; 13 Clinical Pharmacology and Therapeutics Section, Institute for Infection and Immunity, St George’s, University of London, London, UK

**Keywords:** Inflammation, Interleukin-6, Atherosclerosis, Cardiovascular disease, C-reactive protein, Cytokines, Human genetics, Mendelian randomization

## Abstract

**Background:**

Interleukin 6 (IL-6) signaling is being investigated as a therapeutic target for atherosclerotic cardiovascular disease (CVD). While changes in circulating high-sensitivity C-reactive protein (hsCRP) are used as a marker of IL-6 signaling, it is not known whether there is effect heterogeneity in relation to baseline hsCRP levels or other cardiovascular risk factors. The aim of this study was to explore the association of genetically predicted IL-6 signaling with CVD risk across populations stratified by baseline hsCRP levels and cardiovascular risk factors.

**Methods:**

Among 397,060 White British UK Biobank participants without known CVD at baseline, we calculated a genetic risk score for IL-6 receptor (IL-6R)-mediated signaling, composed of 26 variants at the *IL6R* gene locus. We then applied linear and non-linear Mendelian randomization analyses exploring associations with a combined endpoint of incident coronary artery disease, ischemic stroke, peripheral artery disease, aortic aneurysm, and cardiovascular death stratifying by baseline hsCRP levels and cardiovascular risk factors.

**Results:**

The study participants (median age 59 years, 53.9% females) were followed-up for a median of 8.8 years, over which time a total of 46,033 incident cardiovascular events occurred. Genetically predicted IL-6R-mediated signaling activity was associated with higher CVD risk (hazard ratio per 1-mg/dL increment in absolute hsCRP levels: 1.11, 95% CI: 1.06–1.17). The increase in CVD risk was linearly related to baseline absolute hsCRP levels. There was no evidence of heterogeneity in the association of genetically predicted IL-6R-mediated signaling with CVD risk when stratifying the population by sex, age, body mass index, estimated glomerular filtration rate, or systolic blood pressure, but there was evidence of greater associations in individuals with low-density lipoprotein cholesterol ≥ 160 mg/dL.

**Conclusions:**

Any benefit of inhibiting IL-6 signaling for CVD risk reduction is likely to be proportional to absolute reductions in hsCRP levels. Therapeutic inhibition of IL-6 signaling for CVD risk reduction should therefore prioritize those individuals with the highest baseline levels of hsCRP.

**Supplementary Information:**

The online version contains supplementary material available at 10.1186/s12916-022-02446-6.

## Background

Chronic inflammation is an emerging therapeutic target for cardiovascular disease (CVD) [1]. Among pharmacological candidates, agents impacting interleukin (IL)-6 signaling have attracted attention due to converging evidence supporting the relevance of IL-6 in atherosclerosis [2]. Data from the Canakinumab Anti-inflammatory Thrombosis Outcome Study (CANTOS) trial showed that the cardiovascular benefit of IL-1β inhibition with canakinumab was proportional to the reductions in IL-6 and high-sensitivity C-reactive protein (hsCRP) levels [3]. A recent phase 2 trial found that ziltivekimab, a monoclonal antibody directly inhibiting IL-6, effectively and safely reduces biomarkers of inflammation and thrombosis among patients with chronic kidney disease [4]. While indirectly targeting the IL-6 pathway with canakinumab led to hsCRP reductions of 35–40% [3], monthly subcutaneous administration of ziltivekimab resulted in a decrease of hsCRP by 77–92% [4]. However, it remains unknown whether larger hsCRP reductions will translate to greater reductions in CVD risk [4], and the ongoing phase 3 cardiovascular outcomes trial testing ziltivekimab will not be completed before 2025 [5].

Mendelian randomization (MR) leverages genetic variants to investigate the effect of exposures on outcomes and can be applied to explore the therapeutic potential of specific drug targets [6]. The random allocation of genetic variants at conception makes this approach less vulnerable to confounding and reverse causation that can impede causal inference in traditional epidemiological investigations [6, 7]. In this study, we performed MR analyses in 397,060 White British UK Biobank participants to investigate (i) the effect of IL-6 receptor (IL-6R)-mediated signaling on CVD risk in relation to baseline hsCRP levels and (ii) whether the effect of IL-6R-mediated signaling on CVD risk varies across population subgroups stratified by cardiovascular risk factors. These results may inform on patient subgroups for inclusion into interventional trials targeting IL-6 signaling for reducing CVD risk.

## Methods

### Study population

This study follows the reporting recommendations by the STROBE-MR Guidelines (Research Checklist) [8]. Analyses were performed in UK Biobank (application #2532), a prospective cohort study of 502,460 individuals aged 37–73 years recruited between 2006 and 2010. The UK Biobank obtained approval from the Northwest Multi-Center Research Ethics Committee. All participants provided written informed consent. The current analysis was based on White British individuals of European genetic ancestry without known CVD with available genetic, biomarker, and outcome data (Table 1).

**Table 1:** Baseline characteristics of the UK Biobank participants included in our analyses stratified by the median IL-6R-mediated signaling genetic score

Variable	IL-6R-mediated signaling GRS < median		IL-6R-mediated signaling GRS > median		*p*-value
Age, years	59	51–64	59	51–64	0.983
Sex, females	114,383	52.8	114,563	52.9	0.585
CRP, mg/dL	1.28	0.63–2.66	1.45	0.71–2.98	< 2.2 × 10^−16^
SBP, mmHg	137	126–151	137	126–151	0.218
DBP, mmHg	77	84–92	77	84–92	0.755
BMI, kg/m^2^	26.8	24.2–30.0	26.8	24.2–30.0	0.847
eGFR, mL/min/1.73 m^2^	88.3	76.5–100.1	88.2	76.3–100.0	0.062
HbA1c, %	5.36	5.15–5.61	5.38	5.15–5.62	4.1 × 10^−07^
LDL-cholesterol, mg/dL	136.5	114.1–159.9	136.3	113.8–159.8	0.038
HDL-cholesterol, mg/dL	53.9	45.1–64.6	53.8	45.1–64.5	0.131
Lipid-lowering drug use	37,296	17.2	37,742	17.4	0.044
Antidiabetic drug use	6,632	3.0	6,913	3.2	0.014
Antihypertensive drug use	47,587	22.0	47,615	22.0	0.920

The results represent median (interquartile range) or *N* (%). The *p*-values are derived from the Mann–Whitney *U* test for quantitative variables and the chi-square test for binary variables and test the null hypothesis that there is no difference in the listed phenotype by median IL6R signaling genetic risk score (GRS)

### Genetic instruments

The genetic risk score (GRS) for IL-6 receptor (IL-6R)-mediated signaling was created as previously described [9–11] and included 26 variants 300 kB within the *IL6R* gene (clumped at pairwise *r*^*2*^ < 0.1) that were associated with hsCRP, a downstream biomarker of IL-6 signaling (Additional file 1: Table S1, Additional file 2: Fig. S1). As previously described [12], we meta-analyzed a genome-wide association study (GWAS) for hsCRP levels in 204,402 European ancestry individuals (Cohorts for Heart and Aging Research in Genomic Epidemiology (CHARGE) Consortium) [13] with data from 318,279 White British individuals in the UK Biobank [14]. This meta-analysis was performed to maximize the number of genetic variants to be leveraged as instruments for IL-6 signaling, in turn optimizing the power of the MR analysis. We selected variants associated with hsCRP levels (*p* < 5 × 10^−8^) after clumping for linkage disequilibrium at *r*^2^ < 0.1 (1000G European reference panel). We then created a genetic risk score (GRS) for IL-6R-mediated signaling, using association estimates from the CHARGE GWAS as weights for the 26 identified variants (Additional file 1: Table S1). As weights for the GRS were taken from a population that did not overlap with UK Biobank, risk of weak instrument bias related to participant overlap was minimized [15]. This GRS is associated with other biomarkers of upregulated IL-6 signaling as well (lower circulating IL-6 and soluble IL-6R levels), as has been previously described (Additional file 2: Fig. S1) [12].

### Study outcomes

Genetic data were linked to inpatient hospital episode records, primary care data and death registry. The outcome considered was a combined CVD endpoint of incident coronary artery disease, ischemic stroke, peripheral artery disease, aortic aneurysm, and cardiovascular death (codes used to define these outcomes in Additional file 1: Table S2). In sensitivity analyses, we examined an alternative outcome excluding aortic aneurysm, because the disease mechanisms might diverge from those of the other outcomes that are primarily related with atherosclerosis [16].

### Statistical analysis

We used the ratio of coefficients method to perform MR analyses [17]. This represents the association of the GRS with the outcome divided by the association of the GRS with hsCRP [18]. Cox regression was used to estimate association of the score with outcomes, incorporating age, sex, principal components 1 to 10 of genetic ancestry, genotyping chip, kinship, and assessment center as covariates. Linear regression was used to estimate the association of the GRS with hsCRP, incorporating the same covariates. In sensitivity analyses, we excluded individuals with evidence of relatedness within the UK Biobank cohort (kinship coefficient < 0.0884).

To explore the shape of the association between genetically predicted IL-6R-mediated and CVD risk across individuals with varying baseline hsCRP levels, we stratified the population into strata based on residual hsCRP levels, defined as a participant’s hsCRP minus the genetic contribution to hsCRP from the GRS. Stratifying on hsCRP directly would introduce collider bias to distort estimates [19]. For each stratum, we calculated the MR estimate for the association of genetically proxied IL-6R-mediated signaling with the outcome using the ratio of coefficients method [18]. Using a flexible semiparametric framework [20], we then performed a meta-regression of the linear MR estimates obtained for each decile against the median hsCRP value per decile. A fractional polynomial test was used to investigate whether a non-linear model fit this meta-regression better than a linear model. This analysis was performed for both absolute and ln-transformed hsCRP levels. In alternative analyses, we stratified the analyses in centiles of hsCRP rather than deciles.

To investigate if the associations between genetically proxied IL-6R-mediated signaling and CVD vary depending on levels of other cardiovascular risk factors, we performed MR analyses stratified by sex and age, and residual values of body mass index (BMI), cystatin C-based estimated glomerular filtration (eGFR), glycated hemoglobin (HbA1c), low-density lipoprotein cholesterol (LDL-C), and systolic blood pressure (SBP) [19].

Statistical significance for all analyses was set at a two-sided *p*-value < 0.05. Statistical analyses were performed in R (v4.1.1).

## Results

From 502,460 individuals enrolled to the UK Biobank, a total of 397,060 individuals were included in analyses (Additional file 2: Fig. S2, Table 1). Median age at recruitment was 59 years (interquartile range 51–64) and 53.9% of the participants were female. The median hsCRP levels among UK Biobank participants were 1.35 mg/dL (interquartile range 0.67–2.80, Table 1). Levels of hsCRP followed a right-skewed distribution (Additional file 2: Fig. S3) were higher among females and older individuals, correlated positively with BMI, LDL-C, SBP, and HbA1c, and correlated negatively with eGFR (Additional file 2: Fig. S4). The GRS for IL-6R-mediated signaling was associated with hsCRP levels among both female and male participants (Additional file 2: Fig. S5).

**Fig. 1:**
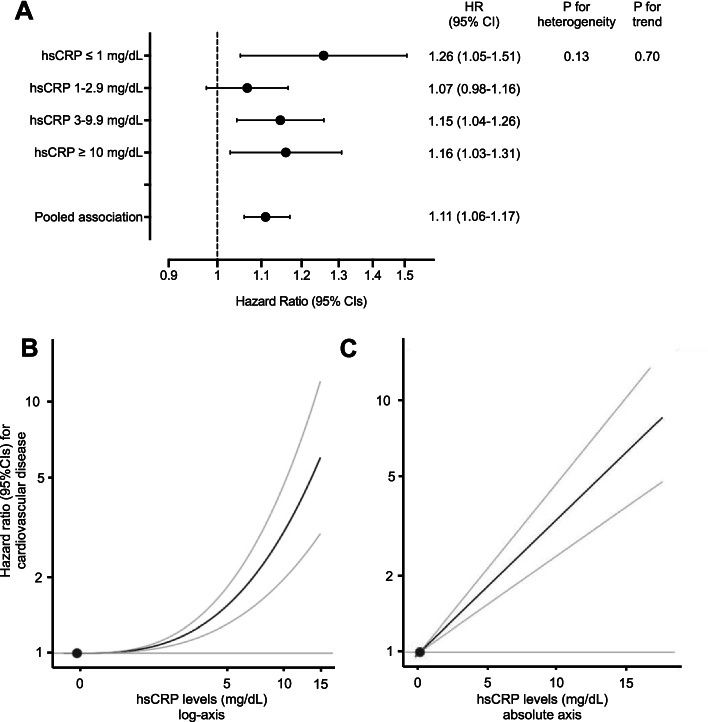
Associations between genetically predicted IL-6R-mediated signaling and risk of incident cardiovascular disease across measured hsCRP levels. **A** Mendelian randomization analyses stratified by baseline hsCRP levels. The hazard ratios are scaled for 1 mg/dL increment in absolute hsCRP levels. The *p*-values for heterogeneity and for trend are derived from the Cochran *Q* statistic and linear meta-regression analyses across deciles of measured hsCRP. **B**, **C** Mendelian randomization analyses of genetically predicted IL6R-mediated signaling and CVD risk across **B** ln-transformed measured hsCRP levels and **C** absolute measured hsCRP levels. For **B**, **C**, results are obtained from fractional polynomial models across associations derived for deciles of measured hsCRP levels. The reference is set to the minimum hsCRP value in the UK Biobank sample (0.08 mg/dL). The *p*-values for non-linearity are 0.001 for ln-transformed hsCRP levels and 0.99 for absolute hsCRP levels. For all graphs, the residual values of hsCRP are used to stratify, as determined in models regressing the genetic risk score for IL-6 signaling on measured hsCRP levels

**Fig. 2:**
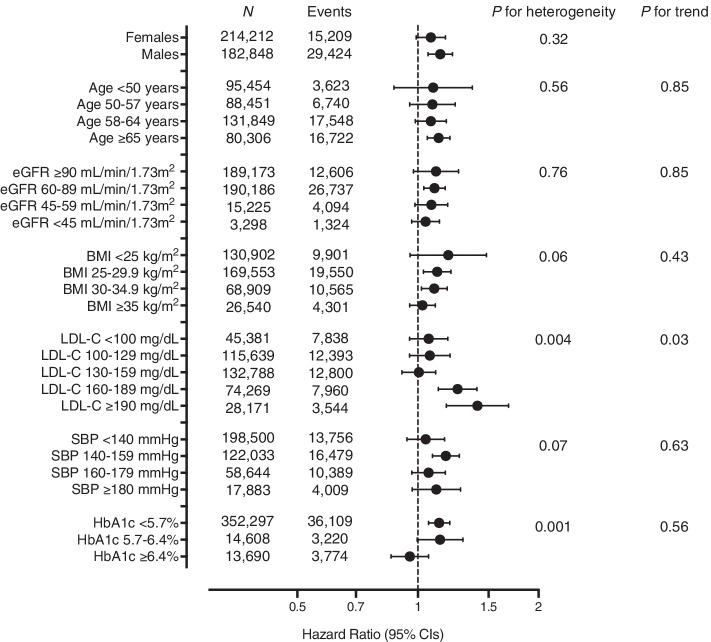
Association between genetically predicted IL-6R-mediated signaling activity and risk of cardiovascular disease across clinically relevant subgroups. The hazard ratios are scaled on 1 mg/dL increment in absolute hsCRP levels. The *p*-values for heterogeneity and for trend are derived from the Cochran *Q* statistic and linear meta-regression analyses across strata of the different measured variables. For all variables except for age and sex, the residual values are used to stratify, as determined in models regressing the genetic score for IL-6 signaling on these variables

Over a median follow-up of 8.8 years (interquartile range 8.1–9.5 years), there were a total of 46,033 incident CVD events. MR analyses identified significant associations between genetically predicted IL-6R-mediated signaling and risk of the composite CVD outcome (hazard ratio per 1-mg/dL increment in absolute hsCRP levels: 1.11, 95% CI: 1.06–1.17, *p* = 6.7 × 10^−5^). This association was similar across individuals with varying baseline hsCRP levels (Fig. 1A) and followed a linear dose–response pattern based with absolute, but not ln-transformed hsCRP levels (Fig. 1B, C). A similar dose–response pattern was observed when stratifying the analyzed UK Biobank population into centiles of hsCRP rather than deciles (Additional file 2: Fig. S6). We observed similar results when excluding aortic aneurysm cases from our main outcome (Additional file 2: Fig. S7). The results also remained materially unchanged when excluding individuals with evidence of relatedness within the UK Biobank (Additional file 2: Fig. S8). Associations were similar in both sexes and across age subgroups. There was no evidence of a trend when stratifying by BMI, eGFR, HbA1c, or SBP (Fig. 2). There was evidence of heterogeneity across subgroups stratified by HbA1c (*p*_*heterogeneity*_ = 0.001) and LDL-C levels (*p*_*heterogeneity*_ = 0.004), with estimates of greater magnitude in individuals with LDL-C levels ≥ 160 mg/dl (*p*_*trend*_ = 0.03).

## Discussion

Our findings are consistent with a linear dose–response relationship between genetically predicted IL-6R signaling and CVD risk in relation to absolute baseline hsCRP levels. For pharmacological purposes, this translates to greater efficacy against CVD of IL-6 signaling inhibition that achieves larger hsCRP reductions.

Our results expand previous genetic data supporting a causal role of IL-6 signaling on atherosclerotic CVD [9, 10, 21] and are consistent with the known effects of IL-6 signaling on increasing CRP generation [22]. In primary prevention cohorts, circulating levels of both IL-6 and CRP have been found to be independently associated with risk of incident CVD [23–25]. Furthermore, the magnitude of cardiovascular risk reduction in the CANTOS trial was directly related to the degree of IL-6 reduction achieved [3]. Taken together, our current genetic findings add to the body of epidemiological and trial evidence supporting a dose response-relationship between IL-6 signaling mediated CRP lowering and CVD risk reduction. In terms of mechanisms underlying such a dose–response relationship, IL-6 is involved in upregulating cellular adhesion molecules at the vessel wall [26], increasing vascular permeability and disrupting endothelial barrier function [27], and promoting vascular smooth muscle growth [28]. It follows that greater absolute reductions in IL-6 signaling, as measured by hsCRP reduction, would confer greater benefit in cardiovascular risk reduction.

When stratifying on other cardiovascular risk factors, there was evidence of greater CVD risk reduction through IL-6R signaling inhibition in individuals with higher LDL-C. This aligns with the notion that inflammation is the result of lipid accumulation in atherosclerotic plaques, and as such, greater benefits may be expected among patients with high baseline LDL-C levels. However, previous genetic analyses have suggested no departure from additive effects on CHD risk when considering genetic proxies for inhibition of IL-6R signaling and pharmacological LDL-C-lowering [11].

A limitation of this work is that it considered European ancestry individuals and may not translate across other ethnic groups. This is particularly relevant as the risk factors and pathophysiological mechanisms underlying CVD may vary across populations of different ethnic ancestry. Furthermore, we considered individuals without CVD at baseline, and it is unclear how these findings will apply to individuals with established CVD, who are at greater absolute risk and therefore likely to be prioritized for treatment. We also did not consider potential adverse effects of perturbing IL-6 signaling, including implications for allergic, autoimmune and infectious disease [10]. Of note, observational and genetic evidence has supported an association of low CRP levels with increased risk of Alzheimer’s disease [29], and further work is required to delineate the dose–response relationship between IL-6 signaling mediated changes in CRP levels and potential adverse outcomes. Finally, these genetic analyses explore small lifelong effects, and may not be directly extrapolated to short-term clinical interventions [6].

## Conclusions

In summary, we find genetic evidence to support that any benefit of pharmacologically inhibiting IL-6 signaling for CVD risk reduction is likely to be proportional to absolute reductions in hsCRP levels. Our results indicate that therapeutic inhibition of IL-6 signaling for CVD risk reduction should prioritize those individuals with the highest baseline levels of hsCRP.

## Supplementary Information

**Additional file 1: Table S1.** Genetic variants included in the genetic risk score for interleukin-6 receptor signaling downregulation and their associations with hsCRP. **Table S2.** Definition of outcomes in the current analysis.

**Additional file 2: Fig. S1.** Associations of genetically predicted IL6 receptor-mediated signaling (measured in 1 unit increment in ln-transformed hsCRP levels) with circulating biomarkers. **Fig. S2.** Selection of study participants. **Fig. S3.** Distribution of (A) absolute and (B) ln-transformed hsCRP levels in the analyzed UK Biobank population. **Fig. S4.** Levels of hsCRP levels by vascular risk factors in the analyzed UK Biobank participants. **Fig. S5.** Levels of high sensitivity C-reactive protein (hsCRP) across deciles of genetic risk score for IL-6 receptor mediated signaling in (A) males and (B) females. **Fig. S6.** Associations between genetically predicted IL-6R-mediated signaling across centiles of measured hsCRP levels and risk of incident cardiovascular disease. **Fig. S7.** Associations between genetically predicted IL-6R-mediated signaling and risk of incident cardiovascular disease across measured hsCRP levels after excluding aortic aneurysm cases from the definition of the outcome. **Fig. S8.** Associations between genetically predicted IL-6R-mediated signaling and risk of incident cardiovascular disease across measured hsCRP levels after excluding individuals with evidence of relatedness within the cohort (kinship coefficient < 0.0884).

## Data Availability

Data from the UK Biobank are available for research purposes following submission of a research proposal. The summary statistics used to generate the IL-6 signaling genetic score are provided in Additional file 1: Table S1.

## Abbreviations

CVD: Cardiovascular disease
GWAS: Genome-wide association study
hsCRP: High-sensitivity C-reactive protein
IL-6: Interleukin-6
IL-6R: Interleukin-6 receptor
MR: Mendelian randomization
