# Supplementary material for: Associations of genetically predicted IL-6 signaling with cardiovascular disease risk across population subgroups
Source: BMC Med. 2022 Aug 11;20:245. doi: 10.1186/s12916-022-02446-6 (PMC9367072; doi:10.1186/s12916-022-02446-6)
Supplement: Supplementary file 1 — Additional file 1: Table S1. Genetic variants included in the genetic risk score for interleukin-6 receptor signaling downregulation and their associations with hsCRP. Table S2. Definition of outcomes in the current analysis. [file 12916_2022_2446_MOESM1_ESM.docx]

**Additional file 1**

**Table S1.** Genetic variants included in the genetic risk score for interleukin-6 receptor signaling downregulation and their associations with hsCRP.

| **SNP** | **chrom** | **bp_hg19** | **effect allele** | **other allele** | **beta CHARGE** | **SE CHARGE** | **p-value CHARGE** | **beta META** | **SE META** | **p-value META** |
| --- | --- | --- | --- | --- | --- | --- | --- | --- | --- | --- |
| rs3766925 | 1 | 154564712 | a | t | -0.0093 | 0.0049 | 0.057701 | -0.0148 | 0.0025 | 2.69E-09 |
| rs78035035 | 1 | 154273429 | a | c | 0.052 | 0.0248 | 0.036014 | 0.0457 | 0.0081 | 1.43E-08 |
| rs112203594 | 1 | 154553430 | a | c | 0.0591 | 0.024 | 0.013797 | 0.0396 | 0.0071 | 2.09E-08 |
| rs3738028 | 1 | 154698817 | a | c | 0.0121 | 0.0047 | 0.010039 | 0.0137 | 0.0023 | 1.19E-09 |
| rs116141616 | 1 | 154416069 | a | g | 0.0614 | 0.0197 | 0.001829 | 0.0387 | 0.0069 | 1.76E-08 |
| rs12406117 | 1 | 154740879 | a | g | 0.0136 | 0.0043 | 0.001563 | 0.0124 | 0.0021 | 3.83E-09 |
| rs144029367 | 1 | 154455249 | t | c | -0.0752 | 0.0185 | 4.81E-05 | -0.0498 | 0.008 | 5.43E-10 |
| rs61806853 | 1 | 154154587 | t | c | 0.0456 | 0.0112 | 4.67E-05 | 0.0437 | 0.005 | 1.23E-18 |
| rs76289529 | 1 | 154516404 | t | c | -0.0715 | 0.0171 | 2.90E-05 | -0.0519 | 0.006 | 4.78E-18 |
| rs6698385 | 1 | 154652572 | a | g | -0.0216 | 0.0051 | 2.28E-05 | -0.035 | 0.0026 | 3.74E-41 |
| rs145262901 | 1 | 154394484 | a | g | -0.0844 | 0.0183 | 3.99E-06 | -0.061 | 0.0102 | 2.38E-09 |
| rs145909430 | 1 | 154391504 | t | c | 0.1396 | 0.0272 | 2.86E-07 | 0.1001 | 0.0082 | 2.86E-34 |
| rs41269913 | 1 | 154461480 | t | c | -0.0779 | 0.0147 | 1.16E-07 | -0.0424 | 0.0058 | 2.28E-13 |
| rs77994623 | 1 | 154505106 | t | c | 0.0332 | 0.0061 | 5.25E-08 | 0.046 | 0.0029 | 1.50E-58 |
| rs183641528 | 1 | 154499328 | a | g | -0.1034 | 0.0178 | 6.29E-09 | -0.0851 | 0.008 | 1.62E-26 |
| rs113580743 | 1 | 154420333 | a | g | 0.0708 | 0.0112 | 2.59E-10 | 0.055 | 0.0055 | 1.09E-23 |
| rs34693607 | 1 | 154661369 | c | g | 0.0368 | 0.0057 | 1.07E-10 | 0.0328 | 0.0026 | 3.83E-36 |
| rs56100876 | 1 | 154496473 | a | g | -0.18 | 0.0262 | 6.41E-12 | -0.117 | 0.0086 | 3.27E-42 |
| rs12735458 | 1 | 154361406 | a | g | 0.126 | 0.0183 | 5.77E-12 | 0.0842 | 0.0092 | 4.53E-20 |
| rs73026617 | 1 | 154369981 | t | c | 0.0474 | 0.0068 | 3.16E-12 | 0.0467 | 0.0034 | 1.69E-42 |
| rs7525477 | 1 | 154394297 | a | g | 0.0382 | 0.0051 | 6.88E-14 | 0.0296 | 0.0023 | 1.35E-38 |
| rs11264224 | 1 | 154568086 | a | c | 0.0465 | 0.0057 | 3.41E-16 | 0.0418 | 0.0028 | 1.60E-49 |
| rs16836054 | 1 | 154462195 | a | g | 0.0453 | 0.0054 | 4.91E-17 | 0.0516 | 0.0028 | 1.51E-75 |
| rs12059682 | 1 | 154579585 | t | c | -0.0441 | 0.0049 | 2.26E-19 | -0.0474 | 0.0025 | 2.11E-77 |
| rs12083537 | 1 | 154381103 | a | g | 0.0643 | 0.0053 | 7.14E-34 | 0.0679 | 0.0026 | 3.03E-156 |
| rs2228145 | 1 | 154426970 | a | c | 0.0899 | 0.0042 | 1.21E-101 | 0.0947 | 0.0021 | 3.00E-307 |

chrom: chromosome; bp_hg19: genomic position according to the GRCh37/hg19 reference genome. SE: standard error; META: meta-analysis

**Table S2.** Definition of outcomes in the current analysis.

| **Outcome** | **N Cases** | **ICD-9** | **ICD-10** | **OPCS** | **Self-report*** |
| --- | --- | --- | --- | --- | --- |
| Coronary artery disease | 34,926 | 410, 411, 412, 414.0, 414.8, 414.9 | I21, I22, I23, I24, I25.1, I25.2, I25.5, I25.6, I25.8, I25.9 | K40, K41, K42, K43, K44, K45, K46, K49, K50.1, K50.2, K50.4, K75 | 20002 |
| Ischemic stroke | 6,929 | 434, 436 | I63, I64 |  | 20002 |
| Peripheral artery disease | 3,902 | 4400, 4402, 4438, 4439 | I70.0, I70.00, I70.01, I70.2, I70.20, I70.21, I70.8, I70.80,  I70.9, I70.90, I73.8, I73.9 | L21.6, L51.3,  L51.6, L51.8,  L52.1, L52.2,  L54.1, L54.4,  L54.8, L59.1,  L59.2, L59.3,  L59.4, L59.5,  L59.6, L59.7,  L59.8, L60.1,  L60.2, L63.1,  L63.5, L63.9,  L66.7 | 20002 |
| Aortic aneurysm | 4,083 | 441 | I71.1-I71.9 | L18, L19, L27, L28 | 20002 |
| Cardiovascular death | 3,179 |  | I chapter |  |  |

* Variable coding in the UK Biobank.

ICD: International Classification of disease. OPCS: Office of Population Censuses and Surveys Classification of Surgical Operations and Procedures Classification of Interventions and Procedures.
