# Supplementary material for: Associations of genetically predicted IL-6 signaling with cardiovascular disease risk across population subgroups
Source: BMC Med. 2022 Aug 11;20:245. doi: 10.1186/s12916-022-02446-6 (PMC9367072; doi:10.1186/s12916-022-02446-6)
Supplement: Supplementary file 2 — Additional file 2: Fig. S1. Associations of genetically predicted IL6 receptor-mediated signaling (measured in 1 unit increment in ln-transformed hsCRP levels) with circulating biomarkers. Fig. S2. Selection of study participants. Fig. S3. Distribution of (A) absolute and (B) ln-transformed hsCRP levels in the analyzed UK Biobank population. Fig. S4. Levels of hsCRP levels by vascular risk factors in the analyzed UK Biobank participants. Fig. S5. Levels of high sensitivity C-reactive protein (hsCRP) across deciles of genetic risk score for IL-6 receptor mediated signaling in (A) males and (B) females. Fig. S6. Associations between genetically predicted IL-6R-mediated signaling across centiles of measured hsCRP levels and risk of incident cardiovascular disease. Fig. S7. Associations between genetically predicted IL-6R-mediated signaling and risk of incident cardiovascular disease across measured hsCRP levels after excluding aortic aneurysm cases from the definition of the outcome. Fig. S8. Associations between genetically predicted IL-6R-mediated signaling and risk of incident cardiovascular disease across measured hsCRP levels after excluding individuals with evidence of relatedness within the cohort (kinship coefficient < 0.0884). [file 12916_2022_2446_MOESM2_ESM.docx]

**Additional file 2**

**Figure S1.** Associations of genetically predicted IL6 receptor-mediated signaling (measured in 1 unit increment in ln-transformed hsCRP levels) with circulating biomarkers.

The error lines correspond to 95% confidence intervals of beta coefficients per 1 ln-hsCRP increment, as derived from fixed effects inverse-variance weighted 2-sample Mendelian randomization analyses. The data for IL-6 and sIL6R are derived from Finnish sample of 8,293 individuals^4^ and a blood proteomics analyses within 3,301 European ancestry individuals in the INTERVAL study,^5^ respectively.

IL-6: interleukin-6; sIL6R: soluble interleukin-6 receptor.

**Figure S2.** Selection of study participants.


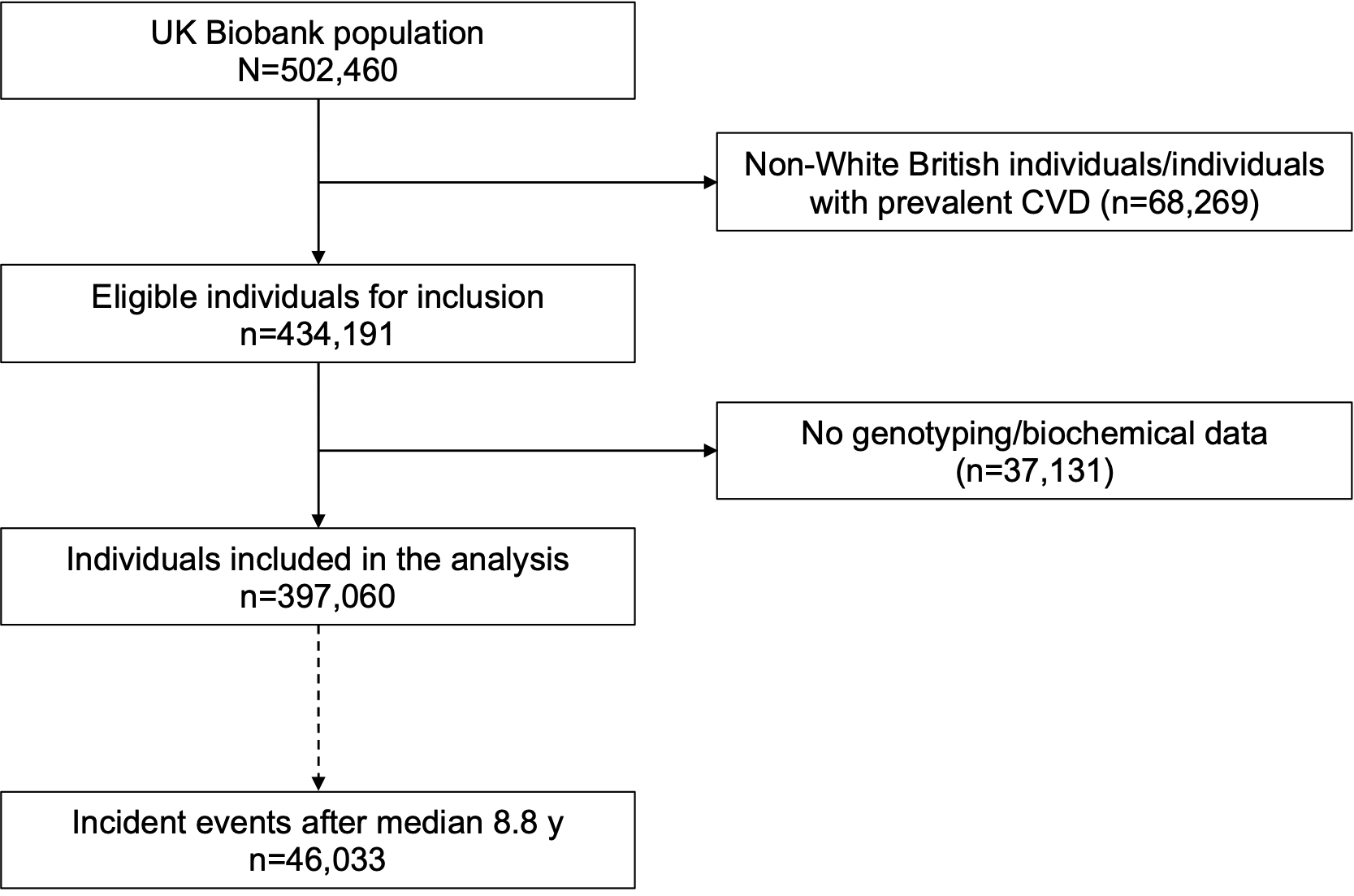


**Figure S3.** Distribution of (A) absolute and (B) ln-transformed hsCRP levels in the analyzed UK Biobank population.


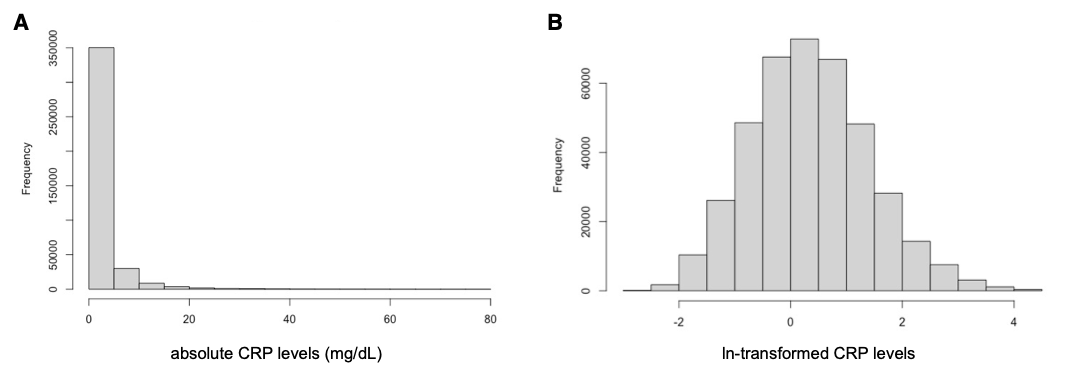


**Figure S4.** Levels of hsCRP levels by vascular risk factors in the analyzed UK Biobank participants.


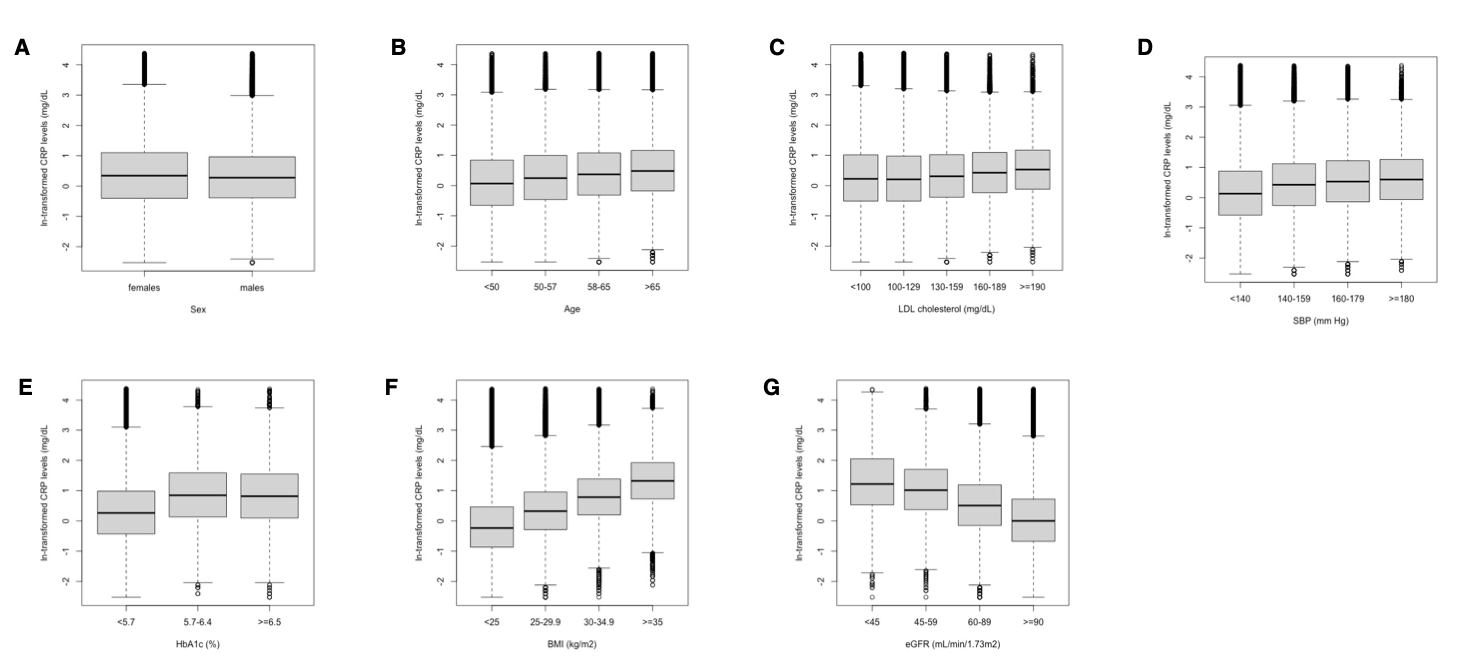


Median levels are presented. The box borders correspond to 25^th^ and 75^th^ percentiles, whereas the error bars to 2.5^th^ and 97.5^th^ percentiles.

**Figure S5.** Levels of high sensitivity C-reactive protein (hsCRP) across deciles of genetic risk score for IL-6 receptor mediated signaling in (A) males and (B) females.


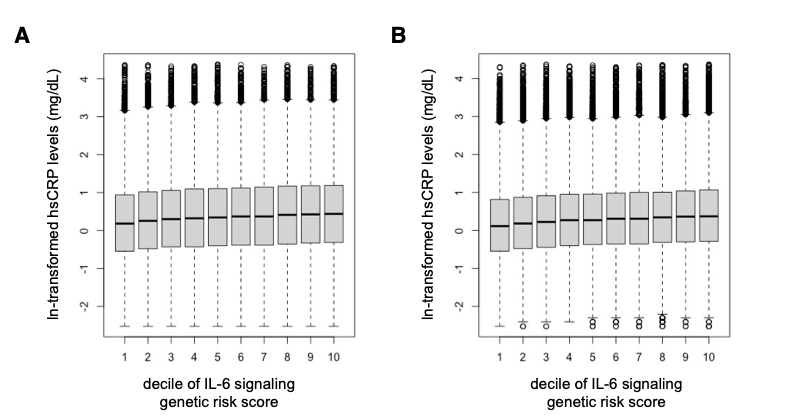


Median levels are presented. The box borders correspond to 25^th^ and 75^th^ percentiles, whereas the error bars to 2.5^th^ and 97.5^th^ percentiles.

**Figure S6.** Associations between genetically predicted IL-6R-mediated signaling across centiles of measured hsCRP levels and risk of incident cardiovascular disease.

Mendelian randomization analyses of genetically predicted IL6R-mediated signaling and CVD risk across (A) ln-transformed measured hsCRP levels and (B) absolute measured hsCRP levels. Results are obtained from fractional polynomial models across associations derived for deciles of measured hsCRP levels. The reference is set to the minimum hsCRP value in the UK Biobank sample (0.08 mg/dL). The p-values for non-linearity are 0.001 for ln-transformed hsCRP levels and 0.99 for absolute hsCRP levels. For all graphs, the residual values of hsCRP are used to stratify, as determined in models regressing the genetic risk score for IL-6 signaling on measured hsCRP levels.

**Figure S7.** Associations between genetically predicted IL-6R-mediated signaling and risk of incident cardiovascular disease across measured hsCRP levels after excluding aortic aneurysm cases from the definition of the outcome.

Mendelian randomization analyses stratified by baseline hsCRP levels. The hazard ratios are scaled for 1 mg/dL increment in absolute hsCRP levels. The p-values for heterogeneity and for trend are derived from the Cochran Q statistic and linear meta-regression analyses across deciles of measured hsCRP.

**Figure S8.** Associations between genetically predicted IL-6R-mediated signaling and risk of incident cardiovascular disease across measured hsCRP levels after excluding individuals with evidence of relatedness within the cohort (kinship coefficient <0.0884).

Mendelian randomization analyses stratified by baseline hsCRP levels. The hazard ratios are scaled for 1 mg/dL increment in absolute hsCRP levels. The p-values for heterogeneity and for trend are derived from the Cochran Q statistic and linear meta-regression analyses across deciles of measured hsCRP.
